# Commentary: COVID-19 and the Path to Immunity

**DOI:** 10.29245/2578-3009/2020/4.1201

**Published:** 2020-11-23

**Authors:** Lisa M. James, Effie-Photini C. Tsilibary, Spyros Charonis, Apostolos P. Georgopoulos

**Affiliations:** 1 Brain Sciences Center, Department of Veterans Affairs Health Care System, Minneapolis, MN, USA; 2 Department of Neuroscience, University of Minnesota Medical School, Minneapolis, MN, USA; 3 Department of Psychiatry, University of Minnesota Medical School, Minneapolis, MN, USA; 4 Center for Cognitive Sciences, University of Minnesota, Minneapolis, MN, USA

## Introduction

A recently published Viewpoint^1^ underscored the importance of T- and B- cell-mediated immune responses to severe acute respiratory syndrome coronavirus 2 (SARS-CoV-2) but omitted to mention the very first step necessary to trigger those responses, namely the formation of a complex between the virus antigen and a suitably matching Human Leukocyte Antigen (HLA) molecule. Here, we discuss the role of HLA in individual variability in immune response to SARS-CoV-2, emphasizing the implications of HLA as potentially underlying sustained symptoms seen in “long-COVID”, as distinguished from the severe, acute COVID-19, which is associated with “stormy” immune response.

## Long-COVID

Increasing evidence documents sustained health effects of SARS-CoV-2, the virus responsible for the ongoing coronavirus-19 (COVID-19) pandemic, in a subset of infected individuals. Symptoms including persistent fatigue, dyspnea, joint pain, and chest pain have been reported in patients up to two months after onset of symptoms^2,3^ as have anecdotal reports of “brain fog”^4^; however, scientific study of “long COVID” is just beginning and the reasons underlying symptom persistence, even among those who experienced mild cases of COVID-19, are not yet understood. Current hypotheses regarding the cause of long-COVID include organ damage resulting from acute SARS-CoV-2 infection^5,6^ and extensive and excessive inflammation^7^. Notably, inadequate ability to develop neutralizing antibodies has also been observed in some COVID patients, leading to a call for further exploration of the interaction between SARS-CoV-2 virus and host immune response^8^. Human leukocyte antigen (HLA), a key player in immune system responses to viral and other foreign antigens, has been implicated in the variability in severity of acute cases of COVID-19^9-11^. We contend that HLA makeup likely also plays a role in sustained symptoms observed in long-COVID and is highly relevant to COVID-19 prevention and intervention.

## HLA plays a critical role in immunity

Due to selective pressure and co-evolution with pathogens, HLA is the most polymorphic region of the human genome, resulting in substantial individual variability in cell-surface HLA molecules. This variability, which has been shown to impact the course of pathogen-related illnesses and has been implicated in treatment of infectious diseases and in vaccine responsiveness^12^, results in differential binding affinity of HLA molecules with foreign antigens. The formation of a complex between a foreign antigen and a suitably matched HLA molecule facilitates T- and B-cell mediated immune responses that are critical to successful host protection. Specifically, the HLA-antigen complex is exported to the cell surface where it is presented to CD8+ cytotoxic T-cells which kill infected cells and CD4+ T-cells to initiate the formation of antibodies by B-cells, resulting in adaptive immunity. However, when this process fails, e.g. because of lack of appropriate HLA makeup, immune responses are deficient resulting in chronic disease due to the persistence of offending antigens or viral pathogens which could not be eliminated^13^.

## Long-COVID and persistent antigens

Since HLA variations affect antigen binding affinity, and, consequently, contribute to variability in B- and T-cell mediated immune response to pathogens, individual variability in HLA could account for the differences in the magnitude of antibody and T-cell response and the wide range of clinical disease severity observed with COVID-19, in otherwise immunocompetent individuals. Indeed, it was found that variability in the regional distribution of the two most frequent HLA haplotypes in Italy overlapped with the variability in COVID-19 incidence and mortality observed in Northern vs Southern Italy^9^. Furthermore, in silico analyses have demonstrated allelic variations in SARS-CoV-2 binding affinity^10,11^. While those studies support associations between HLA and acute COVID-19 severity, we are not aware of any study definitively linking long-COVID to specific HLA genotypes. However, research in other diseases has indeed provided evidence of sustained antigen persistence and chronic symptoms in individuals lacking specific “protective” HLA^13,14^. That is, it has been shown that “protective” HLA suitably bind with and eliminate antigens, protecting against chronic disease whereas the absence of protective HLA contribute to immune-mediated disease. Based on those findings, we suspect that the absence of a sufficient HLA-SARS-CoV-2 antigen match leads to the persistence of SARS viral antigens, thereby contributing to sustained symptoms observed in “long-haulers”, a hypothesis that remains to be investigated.

## Consideration of HLA in COVID prevention and intervention

Numerous alleles are likely to bind with SARS-CoV-2 antigens to facilitate elimination of viral antigens and promote recovery; research in our lab is currently underway to identify SARS-CoV-2-protective HLA alleles as such findings bear implications for SARS-CoV-2 prevention and treatment. With regard to other infectious diseases it is well-established that HLA variation is implicated in both vaccine responsiveness and treatment response. For example, administration of a vaccine may not result in antibody production and immunological memory, the very goal of vaccination, in those without suitable HLA makeup – i.e., in the absence of HLA that sufficiently bind with vaccine antigens. Indeed, in individuals lacking specific HLA, vaccine-derived antigens have been found in the serum of individuals affected with chronic conditions decades after vaccination^14^. It stands to reason that HLA is likely to similarly play a role in vaccine response aimed at prevention of COVID-19. That is, a small subset of individuals who lack HLA composition that permits successful binding to the SARS-CoV-2 antigen and stimulation of an immune response are unlikely to benefit from vaccination. Similarly, HLA reflects an important consideration with regard to effective treatment of COVID-19, particularly among the long-haulers. Since those with sustained symptoms are presumably unable to mount a sufficient immune response to eliminate the virus, immunotherapy aimed directly at elimination of the antigens (e.g, via monoclonal or polyclonal antibodies) represents a promising treatment avenue for the subset of COVID-19 cases with sustained symptoms in particular, as demonstrated in other conditions^14^.

## Conclusion

Taken together, HLA is likely implicated in individual variability in disease course and chronicity as well as in prevention and intervention effectiveness. The blind spot created in the field by lack of consideration of variation in HLA with respect to binding affinity of SARS-CoV-2 will hamper efforts at forecasting disease outcomes, developing effective interventions, and creating durable protection. Rather, identification of specific epitopes in the SARS-CoV-2 spike glycoprotein that bind with high affinity to HLA Class II alleles^11^ would prove valuable in understanding adaptive immunity to COVID-19 and advancing successful interventions by developing vaccines based on such HLA-related SARS-CoV-2 epitopes.
